# The effect of epiregulin on epidermal growth factor receptor expression and proliferation of oral squamous cell carcinoma cell lines

**DOI:** 10.1186/1475-2867-14-65

**Published:** 2014-11-18

**Authors:** Darren Chyi-Hsiang Kong, Kenneth Yee Choy Chew, Eng Lai Tan, Suan Phaik Khoo

**Affiliations:** International Medical University (IMU), Bukit Jalil, 57000 Kuala Lumpur, Malaysia; Monash University Malaysia, 47500 Bandar Sunway, Selangor Darul Ehsan, Malaysia; School of Pharmacy, International Medical University (IMU), Bukit Jalil, 57000 Kuala Lumpur, Malaysia; School of Dentistry, International Medical University (IMU), Bukit Jalil, 57000 Kuala Lumpur, Malaysia

**Keywords:** Oral squamous cell carcinoma, Epiregulin, Epidermal growth factor receptor, ErbB4

## Abstract

**Background:**

Epiregulin (EPR) is a novel member of the epidermal growth factor (EGF) family. It has been shown to promote wound healing in oral epithelium, enhance proliferation of other epithelial tissues, and is involved in several epithelial-related malignancies such as colorectal, lung, and bladder carcinoma. More recently, EPR transcripts were found to be high in a study on archival oral squamous cell carcinoma (OSCC) specimens. This implies that EPR may be responsible for the progression of OSCC. The aim of this was to elucidate the effects of EPR on (i) cell morphological changes, (ii) cell proliferation and (iii) receptor expression of the H-series OSCC cell lines.

**Methods:**

The clinicopathological origin and the expression of the epidermal growth factor receptor (EGFR) and ErbB4 receptors of the H-series cell lines were initially characterised. Based on these parameters, two of the H-series cell lines, namely H103 and H357 were selected for downstream experiments. The cell lines were treated with 1 ng/ml, 10 ng/ml, and 20 ng/ml of EPR for 24 and 48 hours in all subsequent experiments. Untreated cells acted as the control which was used for comparison with each treated group. The cell morphological changes, cell proliferation and receptor expression of the OSCC cell lines were evaluated using phase contrast microscopy, 5-bromo-2’-deoxy-uridine (BrdU) assays and flow cytometry respectively. The results were compared and analysed using the student t-test.

**Results:**

There were no appreciable morphological changes in the cells regardless of the dose of EPR tested nor between the different timelines. There were no significant changes in cell proliferation after EPR treatment. As for the effect of EPR on receptor expression, 20 ng/ml of EPR significantly reduced the density of EGFR expression (p value = 0.049) in the H103 cell line after the 24-hour treatment. No other statistically significant changes were detected.

**Conclusions:**

The results show that EPR had no effect on the morphology and proliferativity of OSCC cells. However, the significant decline in EGFR expression after EPR treatment suggests that EPR might play an important role in the regulation of EGFR expression and hence OSCC progression.

## Introduction

Oral cancer is the eighth most common cancer in the world [1], with oral squamous cell carcinomas (OSCCs) making up the majority of oral cancers [2]. Diagnosis is often made when the cancer is in the late stages of malignancy, and this accounts for the poor prognosis of OSCC despite advances in treatment options and protocols for oral cancer management [3–8]. The contributors to the poor prognosis of OSCC are probably multifactorial including environmental exposures especially tobacco smoking and alcohol consumption, genetic susceptibility [3, 9], and molecular influences [10–13] such as growth factors and their receptors.

The epidermal growth factor (EGF) family consists of 13 polypeptide members: EGF, transforming growth factor-alpha (TGF-α), amphiregulin (AR), heparin-binding EGF-like growth factor (HB-EGF), betacellulin (BTC), epiregulin (EPR), epigen (EPG), and six neuregulins (NRGs): NRG-1 to NRG-6 [14–20]. Endogenously, these ligands are found in the form of transmembrane pro-ligands which are proteolytically cleaved to release the mature soluble form which will then proceed to interact with EGF receptors [21]. There are four types of EGF receptors: epidermal growth factor receptor (EGFR), ErbB2, ErbB3, and ErbB4. The binding of EGF ligand members to these EGF receptors can lead to dimerisation of the receptors which subsequently initiates downstream intracellular signalling cascades ultimately resulting in their mitogenic effects [14, 16, 22]. An array of transcription factors are activated through these signalling cascades, producing cellular proliferation, differentiation, apoptosis, chemotaxis, and migration [23–27].

Based on previous studies, the EGF family may play a role in OSCC progression due to its multitude of physiological and malignant effects in human tissues and mice models [28–50]. For example, a study on the saliva of OSCC patients demonstrated raised EGF levels post-surgery, which may indicate OSCC regeneration secondary to tumour tissue injury thus implying a role of EGF in the development of OSCC. In other studies, EGF and HB-EGF were shown to promote cell migration and invasion in OSCC cell lines via enhancement of matrix metalloproteinase (MMP) activity [51, 52]. The EGF family ligands – AR, EPG, EPR, TGF-α and HB-EGF – were also shown to be expressed at high levels in an OSCC cell line, which suggests that the EGF family may play an important role in OSCC progression [51]. Another study demonstrated that higher levels of TGF-α was correlated with lower tumour differentiation, incriminating that TGF-α may somehow be responsible for OSCC differentiation or de-differentiation [53]. Due to the paucity of studies and the limited nature of existing studies, the relationship between the EGF family and OSCC remains unclear and it can only be speculated that the EGF family regulates OSCC proliferation, migration, invasion, and differentiation, based on known interactions between the EGF family, the EGF receptors, and the outcomes of their intracellular signalling cascades.

Epiregulin is one of the novel members of the EGF family, and was initially purified from a conditioned medium of the NIH3T3/clone T7 mouse fibroblast-derived tumour cell line [15]. Epiregulin binds directly to EGFR and ErbB4 [54]. Activation of EGFR by EPR can lead to activation of downstream Ras-Raf-mitogen activated protein kinase (MAPK)-extracellular signal-regulated kinase (ERK) as well as the phosphatidylinositol-3-kinase (PI3K)-Akt pathways to increase cellular proliferation [55–58]. On the other hand, it is unclear whether activation of ErbB4 by EPR stimulates increased cellular proliferation although one study did show that another EGF family member, NRG-1β, increased proliferation by coupling MAPK to ErbB4 [59]. Physiologically, it has been shown to promote wound healing in the oral cavity by stimulating cellular migration of gingival epithelial and fibroblast cells, as well as promoting the proliferation and repopulation of injured gingival epithelium [35]. Epiregulin also stimulates proliferation of keratinocytes, vascular smooth muscle cells (VSMCs), corneal epithelium, renal proximal tubular cells (RPTCs), and fibroblasts [55, 60–62]. In terms of EPR’s malignant potential, an up-regulation of EPR expression was found in some human cancers such as bladder cancer and malignant fibrous histiocytoma (MFH) [63, 64]. Additionally, EPR transcripts were shown to be expressed in bladder, lung, kidney, colon, and epidermoid carcinoma cell lines [65].

In 2008, Shigeishi *et al.*[34] showed that in archival OSCC tissue specimens, EPR messenger ribonucleic acid (mRNA) expression was found to be significantly greater when compared to normal gingivae and dysplastic epithelium, and this was correlated with poor patient clinical outcome. These findings, together with the fact that EPR is involved in a wide range of human cancers, suggest that the poor clinical outcome may be caused by the influence of EPR on tumour progression in terms of proliferation, migration, and invasion. This has provided some insight into the possible role of EPR in oral cancer.

In order to determine whether EPR has a potential effect in OSCC tumour progression *in vitro*, the current study reports the effect of EPR on cell morphology, cell proliferation, and cellular expression of EGFR and ErbB4 receptors in OSCC cell lines.

## Results

### Selection of cell lines for downstream experiments

#### Clinicopathological considerations

Out of the seven cell lines, H103 and H357 share similar clinicopathological characteristics with regards to the sex and Site (S)-Size (T)-Nodal metastasis (N)-Distant metastasis (M)-Pathology (P) (STNMP) grades of the patients (see Table 1) [66]. However, these two cell lines differ with regards to the patients’ age.

**Table 1:** **Clinicopathological characteristics of the H-series cell lines**

Cell line	Nationality	Age	Sex	Site ^a^ (S)	Size (T)	Nodal metastasis (N)	Distant metastasis (M)	Pathology ^b^ (P)	STNMP grade ^c^
H103	British	32	M	T	< 20	-	-	W	I
H157	British	84	M	BM	20-40	+	-	W	II
H314	British	82	M	FOM	20-40	+	-	M	II
H357	British	74	M	T	< 20	-	-	W	I
H376	British	40	F	FOM	20-40	+	-	W	III
H400	British	55	F	AP	20-40	-	-	M	II
H413	British	53	F	BM	20-40	-	-	M	II

^a^Site: T, tongue; BM, buccal mucosa; FOM, floor of mouth; AP, alveolar process.

^b^Pathology: W, well-differentiated; M, moderately-differentiated; P, poorly-differentiated.

^c^STNMP grade: prognostic indicator for OSCC with 51.5%, 40.7%, 21.6% and 8.3% 5 year survival for patients with a stage I, II, III or IV tumours, respectively.

#### EGFR and ErbB4 receptors screening

**Figure 1:**
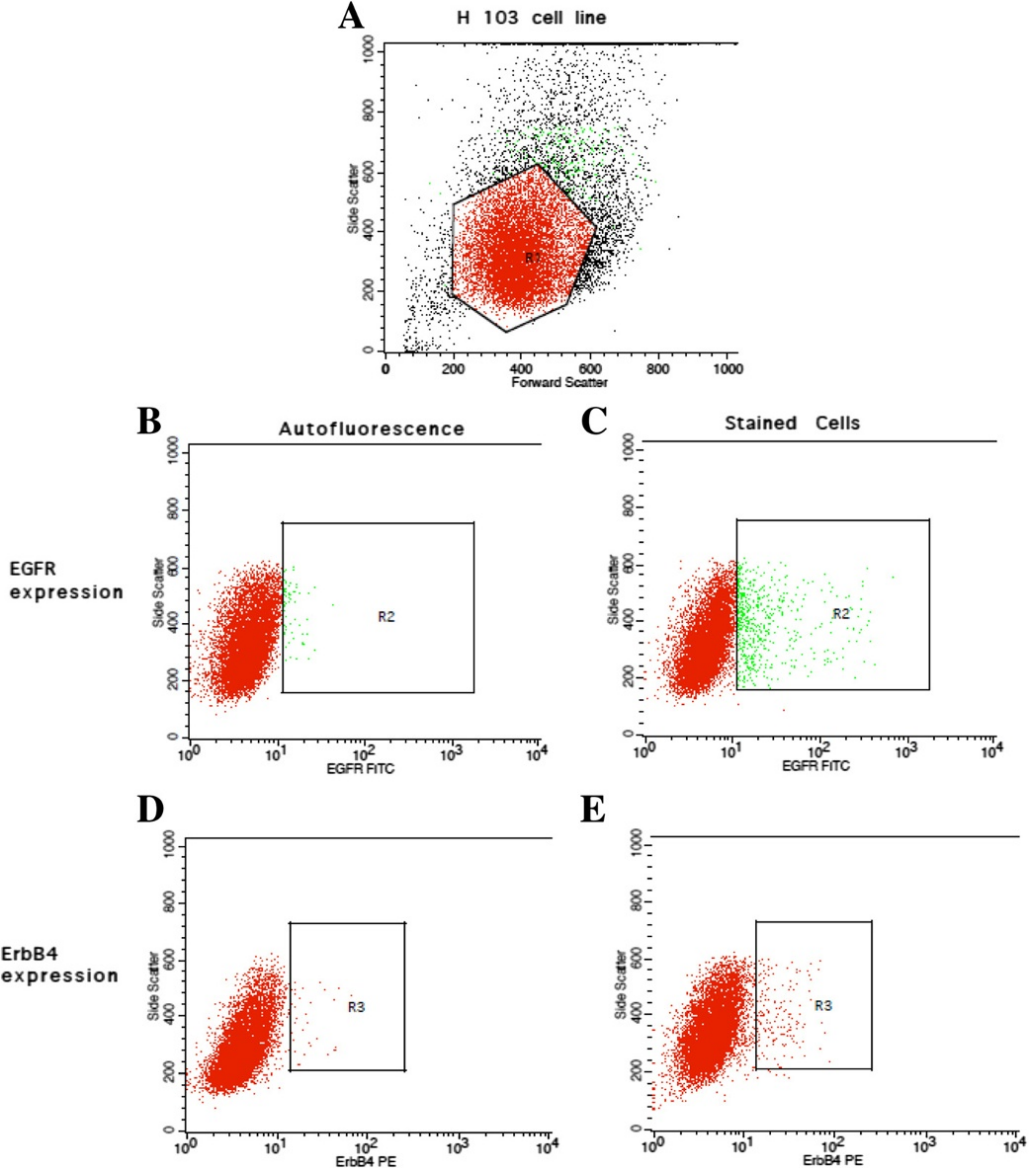
**Flow cytometric output for the EGFR and ErbB4 receptors screening in the H103 cell line.** The true percentage of cells expressing EGFR or ErbB4 receptors were calculated by subtracting the autofluorescence of the unstained cells (B and D) from the fluorescence of the cells stained with the anti-EGFR-FITC or anti-ErbB4-PE antibodies (C and E) respectively. **A** The polygonal box, R_1_ represents the gating of the cells based on the forward and side scatter profiles to exclude cellular debris and doublet cells. A total of 10,000 gated events were acquired for analysis. **B** The green dots within the R_2_ box represent the autofluorescence of unstained cells. This autofluorescence was subtracted from the fluorescence detected in C. **C** The green dots within the R_2_ box represent the fluorescence of cells after staining with anti-EGFR-FITC antibodies. **D** The pink dots within the R_3_ box represent the autofluorescence of unstained cells. This autofluorescence was subtracted from the fluorescence detected in E. **E** The pink dots within the R_3_ box represent the fluorescence of cells after staining with anti-ErbB4-PE antibodies.

**Figure 2:**
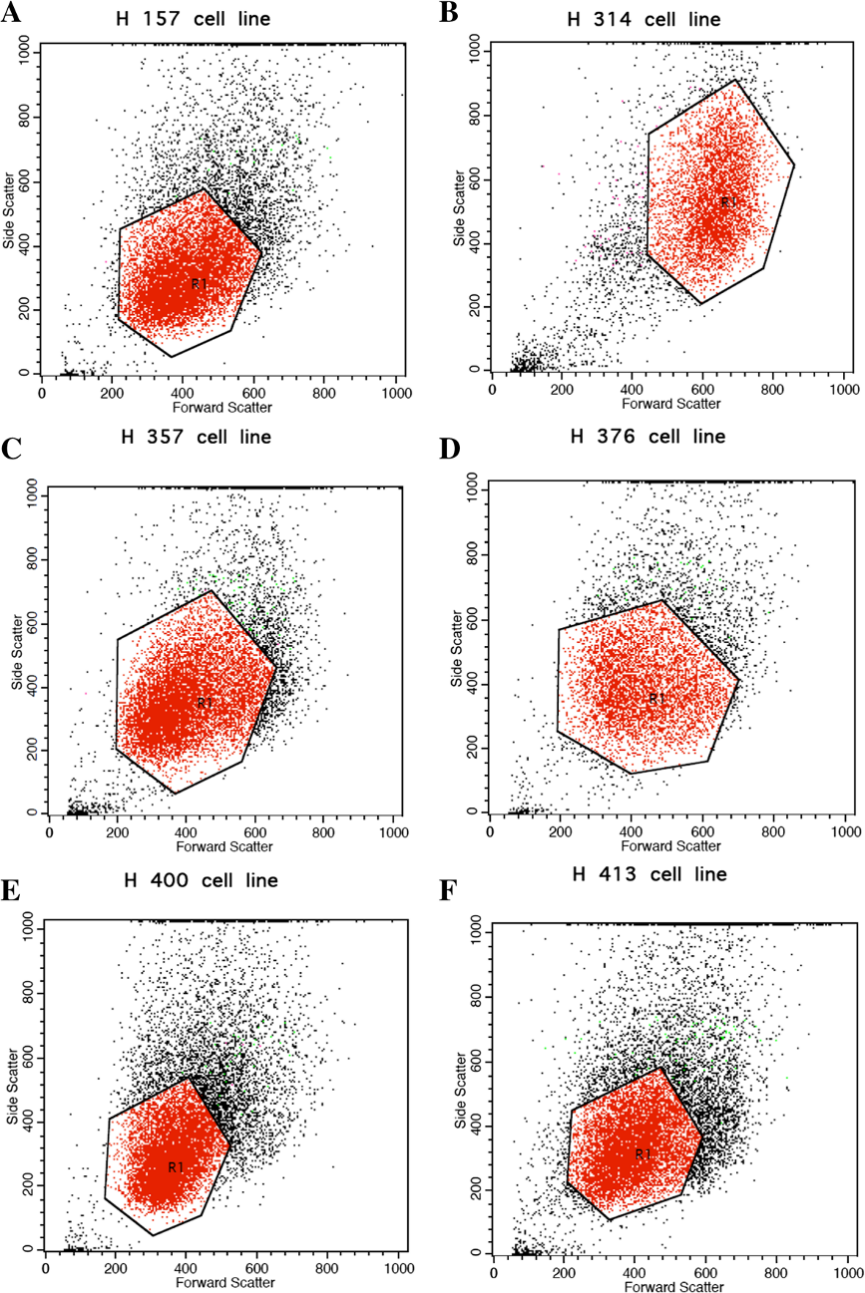
**Flow cytometric output for the EGFR and ErbB4 receptors screening in the other H-series cell lines.** The polygonal R_1_ boxes in **A–F** (cell lines are **A** H157, **B** H314, **C** H357, **D** H400, and **E** H413) represents the gating of the cells based on the forward and side scatter profiles to exclude cellular debris and doublet cells. A total of 10,000 gated events were acquired for analysis for each cell line. Similar analyses to obtain the true percentage of cells expressing EGFR and ErbB4 receptors were performed as per Figure 1.

Figures 1 and 2 show the flow cytometric outputs for the EGFR and ErbB4 receptor screening. The results of the screening are shown in Table 2.

**Table 2:** **Receptor status for the H-series of cell lines**

Cell line	Receptor status			
	EGFR expression (%)	Positivity	ErbB4 expression (%)	Positivity
**H103**	0.72	+	0.19	+
**H157**	0.91	+	0.12	+
**H314**	0.47	+	0.03	+
**H357**	2.34	+	0.00	-
**H376**	1.94	+	0.10	+
**H400**	1.84	+	0.53	+
**H413**	7.74	+	0.27	+

With regards to the EGFR and ErbB4 expression profiles, all the H-series cell lines expressed both the EGFR and ErbB4 receptors. However, the ErbB4 expression of H357 was negative.

Taking the patients’ clinicopathological characteristics as well as the presentation of the receptors into consideration, the H103 and H357 cell lines were selected. The H357 cell line was selected to serve as a negative control in the setting of downstream experiments due to its ErbB4 negativity.

### Determination of cell morphology

#### H103 cell line

**Figure 3:**
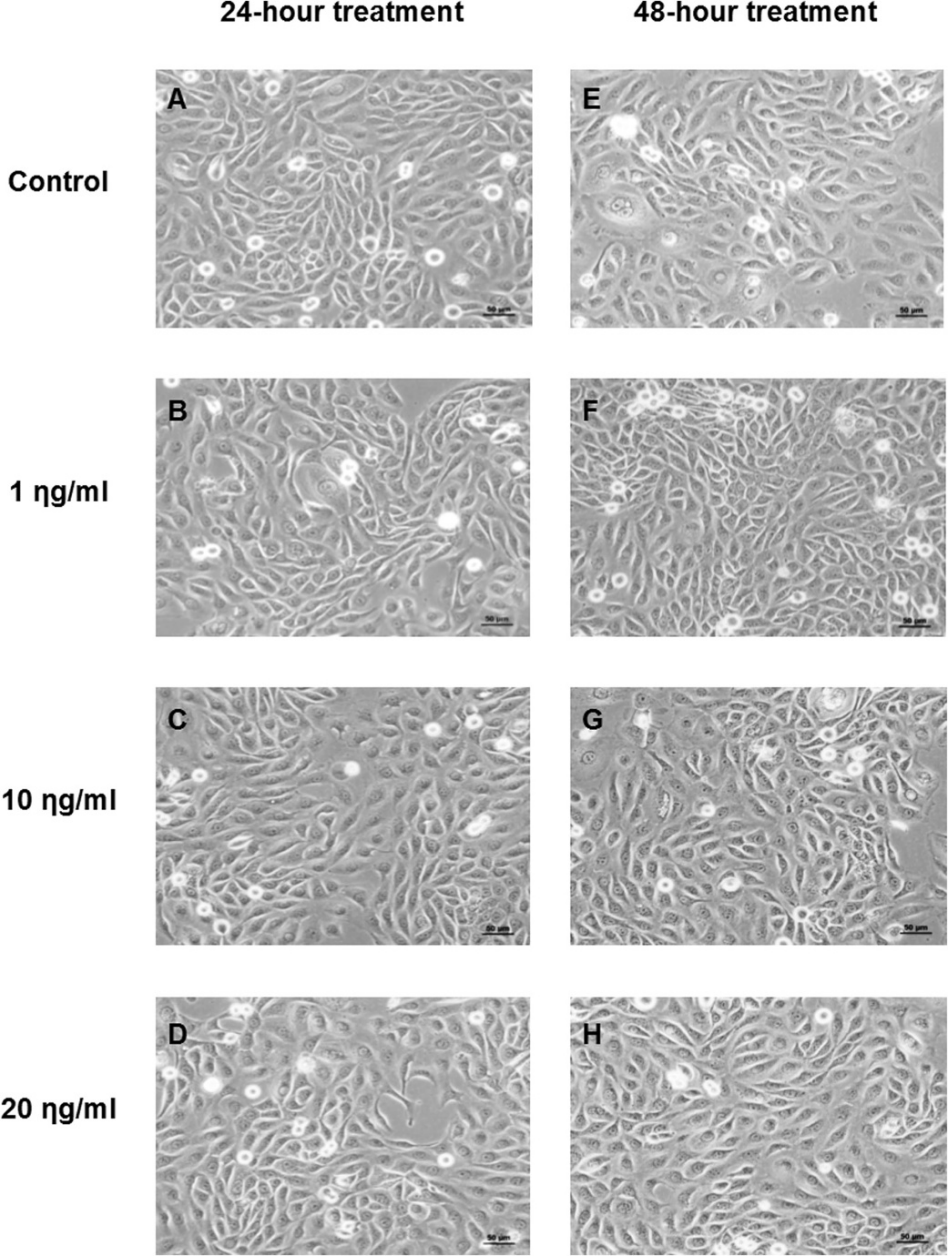
**Cell morphology of the H103 cell line after 24- and 48-hour treatment. A** and **E** Untreated control. **B** and **F** Treated with 1 ηg/ml EPR. **C** and **G** Treated with 10 ηg/ml EPR. **D** and **H** Treated with 20 ηg/ml EPR. The cells were assessed under low magnification (20x) phase contrast microscopy for any changes in morphology between the treated groups and the untreated control within each timeline, between the treated groups and between the two timelines of treatment. The cells were assessed in terms of size, shape, nuclear and cytoplasmic changes.

Figures 3A-D show the morphology of cells after 24-hour treatment with EPR. The cell line displayed a heterogeneous population of small to large cells with a spindle-like appearance. There were no differences in morphology between the treated groups (regardless of the dose of EPR) and the untreated control. Figures 3E-H show the morphology after 48-hour treatment. The cell line also displayed similar morphology as those after 24-hour treatment. Not only were there no morphological changes between the groups within each timeline, there were also no differences between the treated groups (regardless of the dose of EPR) and the untreated control. No differences in morphology between the two timelines of treatment were found.

#### H357 cell line

**Figure 4:**
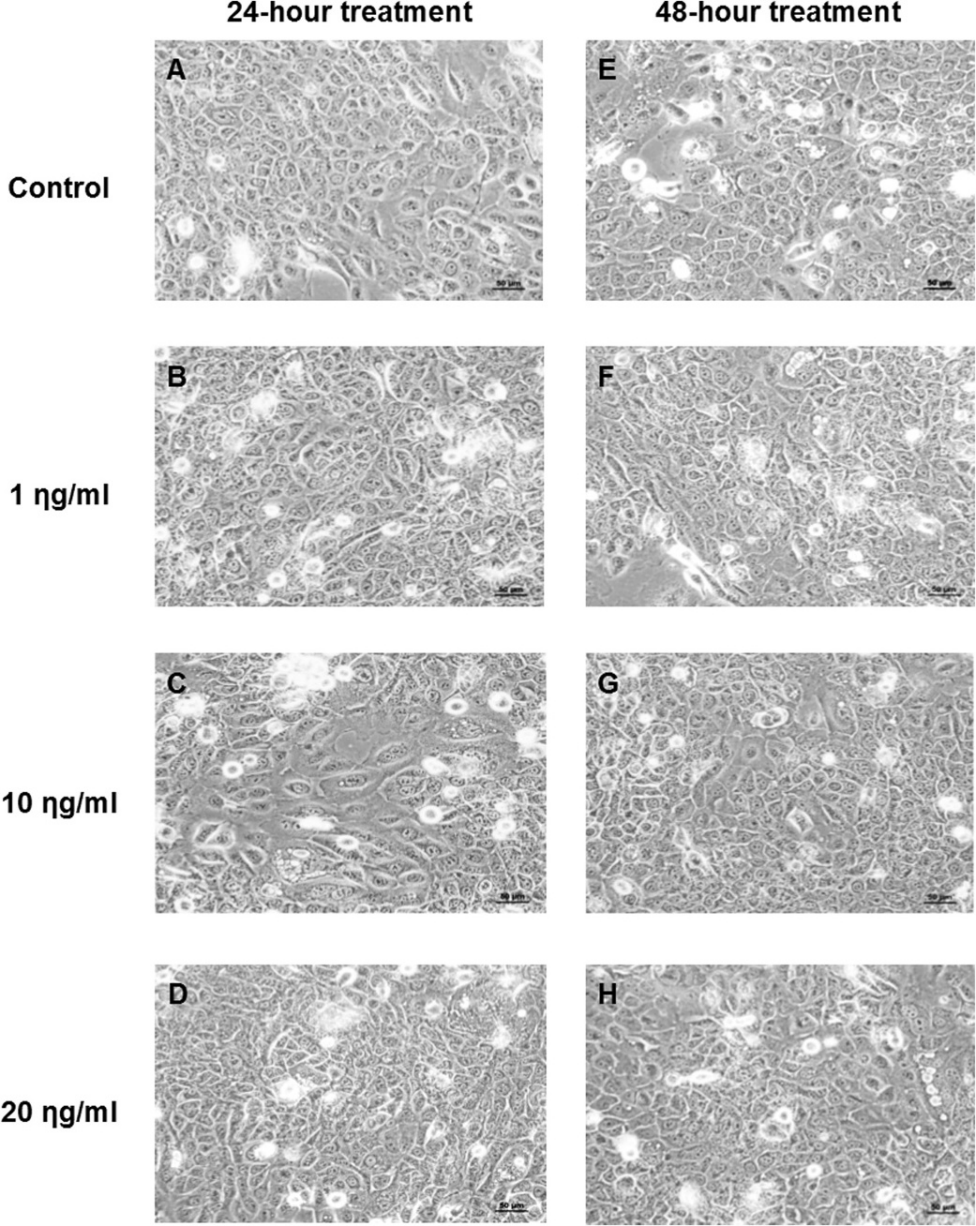
**Cell morphology of the H357 cell line after 24- and 48-hour treatment. A** and **E** Untreated control. **B** and **F** Treated with 1 ηg/ml EPR. **C** and **G** Treated with 10 ηg/ml EPR. **D** and **H** Treated with 20 ηg/ml EPR. The cells were assessed under low magnification (20x) phase contrast microscopy for any changes in morphology between the treated groups and the untreated control within each timeline, between the treated groups and between the two timelines of treatment. The cells were assessed in terms of size, shape, nuclear and cytoplasmic changes.

Figures 4A-D show the morphology of cells after 24-hour treatment. The cell line displayed a heterogeneous population of small to large cells with rounded appearance. There were no differences in morphology between the treated groups (regardless of the dose of EPR) and the untreated control. Figures 4E-H show the morphology after 48-hour treatment. The cell line also displayed similar morphology as 24-hour treatment. Not only were there no morphological changes between the groups within each timeline, there were also no differences between the treated groups (regardless of the dose of EPR) and the untreated control. No differences in morphology between the two timelines of treatment were found.

The findings showed that there were no morphological changes in the cells after treatment with EPR.

### Determination of cell proliferation

The proliferation of the OSCC cells was measured based on (i) cell count and (ii) 5-bromo-2’-deoxy-uridine (BrdU) assay. In order to assess any changes in cell proliferation after EPR treatment, we compared the cell count/absorbance of cells between each EPR-treated group, namely 1 ng/ml, 10 ng/ml and 20 ng/ml, with the control.

### (i)Proliferation based on cell count

#### H103 cell line

**Figure 5:**
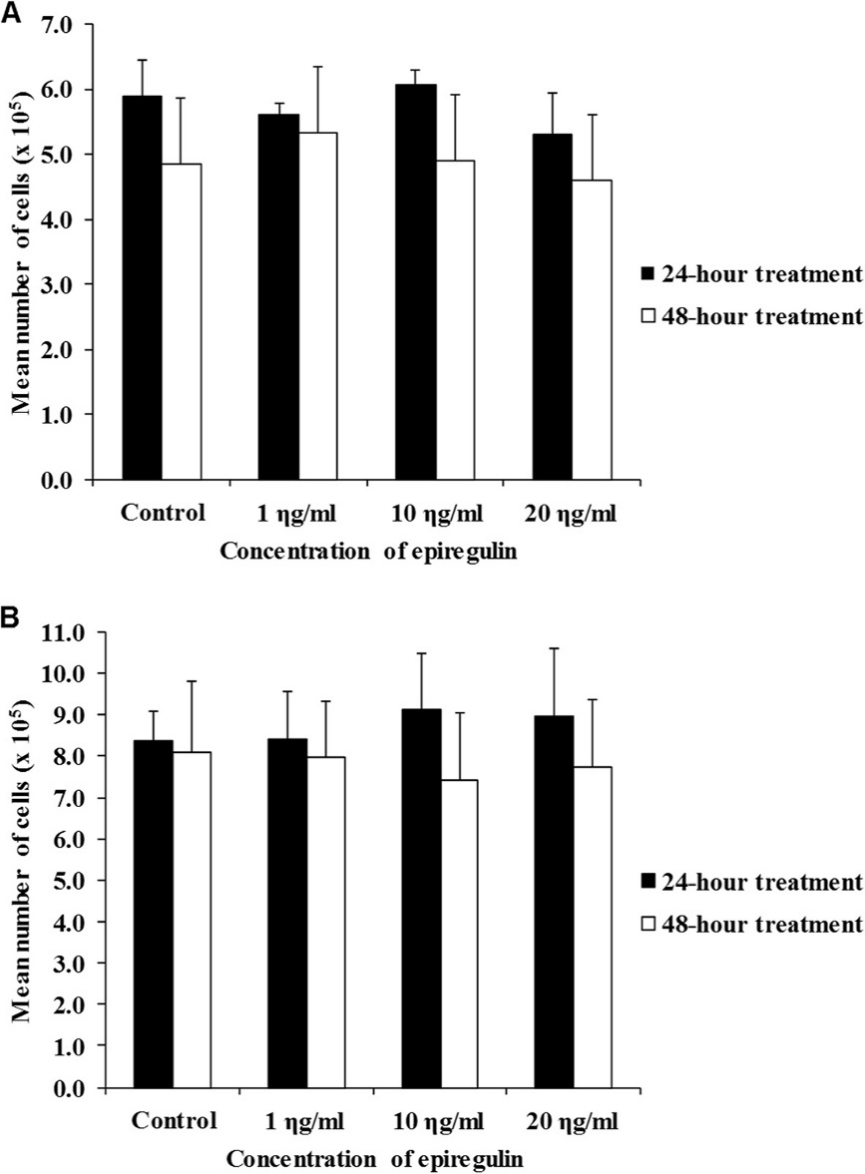
**Cell counts. A** H103 cell line after 24- and 48-hour treatment. **B** H357 cell line after 24- and 48-hour treatment. Bars represent mean ± standard error of mean of a triplicate of experiments. The findings showed no statistically significant differences in cell counts after treatment with EPR.

Figure 5A shows a marginal increase in cell counts compared to the control, when 10 ng/ml of EPR was added to the cell line and left for 24 hours. There were also similar increases in cell counts when 1 ng/ml and 10 ng/ml of EPR were added to the cell lines and left for 48 hours. These increases however, were not statistically significant.

#### H357 cell line

Figure 5B shows marginal increase in cell counts compared to the control in all treated groups (regardless of the dose of EPR) when EPR was added to the cell line and left for 24 hours. However, there were no increases in cell counts in all treated groups (regardless of the dose of EPR) when EPR was added to the cell line and left for 48 hours. These increases however, were not statistically significant.

The findings showed that there were no significant changes in the cell counts after treatment with EPR.

### (ii)Proliferation based on BrdU assay

#### H103 cell line

**Figure 6:**
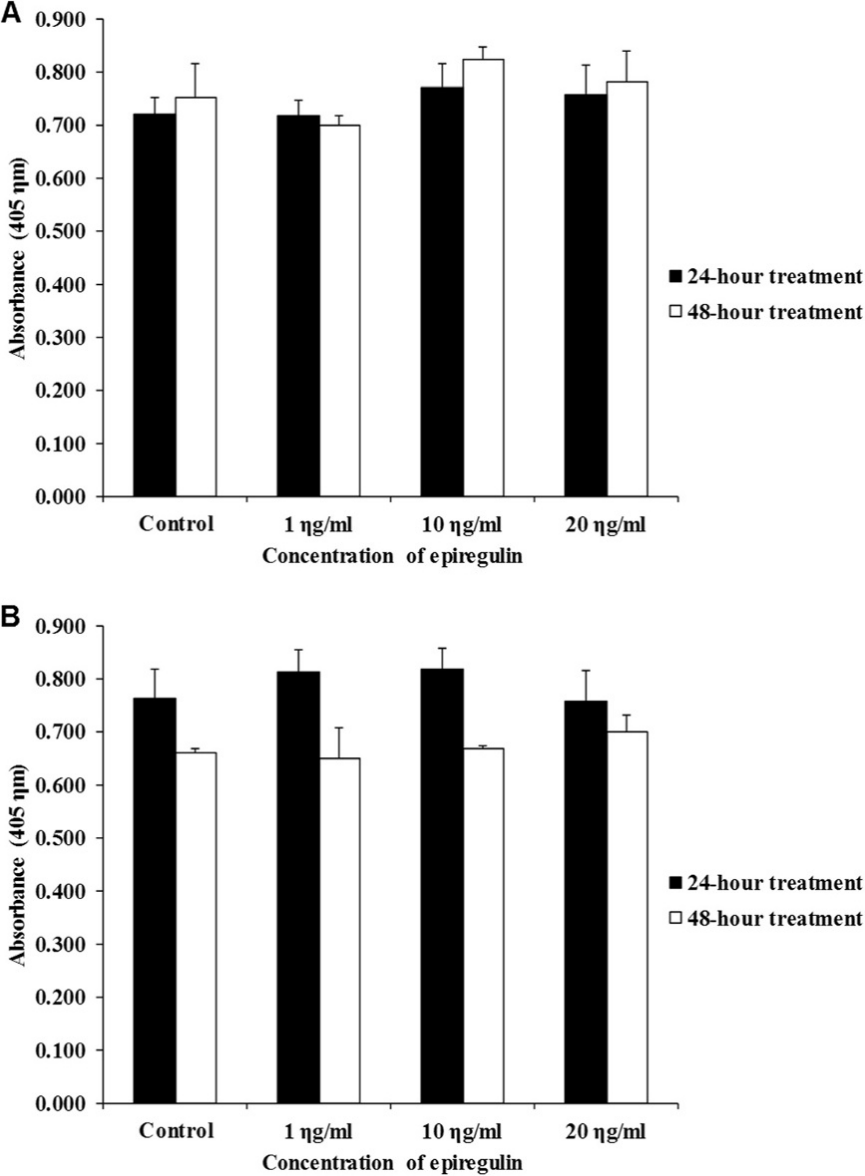
**BrdU assay. A** H103 cell line after 24- and 48-hour treatment. **B** H357 cell line after 24- and 48-hour treatment. Bars represent mean ± standard error of mean of a triplicate of experiments. The findings showed no statistically significant differences in absorbance after treatment with EPR.

Figure 6A shows marginal increases in cell proliferation compared to the control, when 10 ng/ml and 20 ng/ml of EPR were added to the cell line and left for 24 and 48 hours. These increases however, were not statistically significant.

#### H357 cell line

Figure 6B shows marginal increases in cell proliferation compared to the control, when 1 ng/ml and 10 ng/ml of EPR was added to the cell line and left for 24 hours. There were also marginal increases in cell proliferation compared to the control when 10 ng/ml and 20 ng/ml of EPR were added to the cell line and left for 48 hours. These increases however, were not statistically significant.

The findings of both the cell counts and BrdU assays showed no significant changes after EPR treatment. This suggests that EPR does not have an effect on cell proliferation.

### EGFR and ErbB4 receptors expression

**Figure 7:**
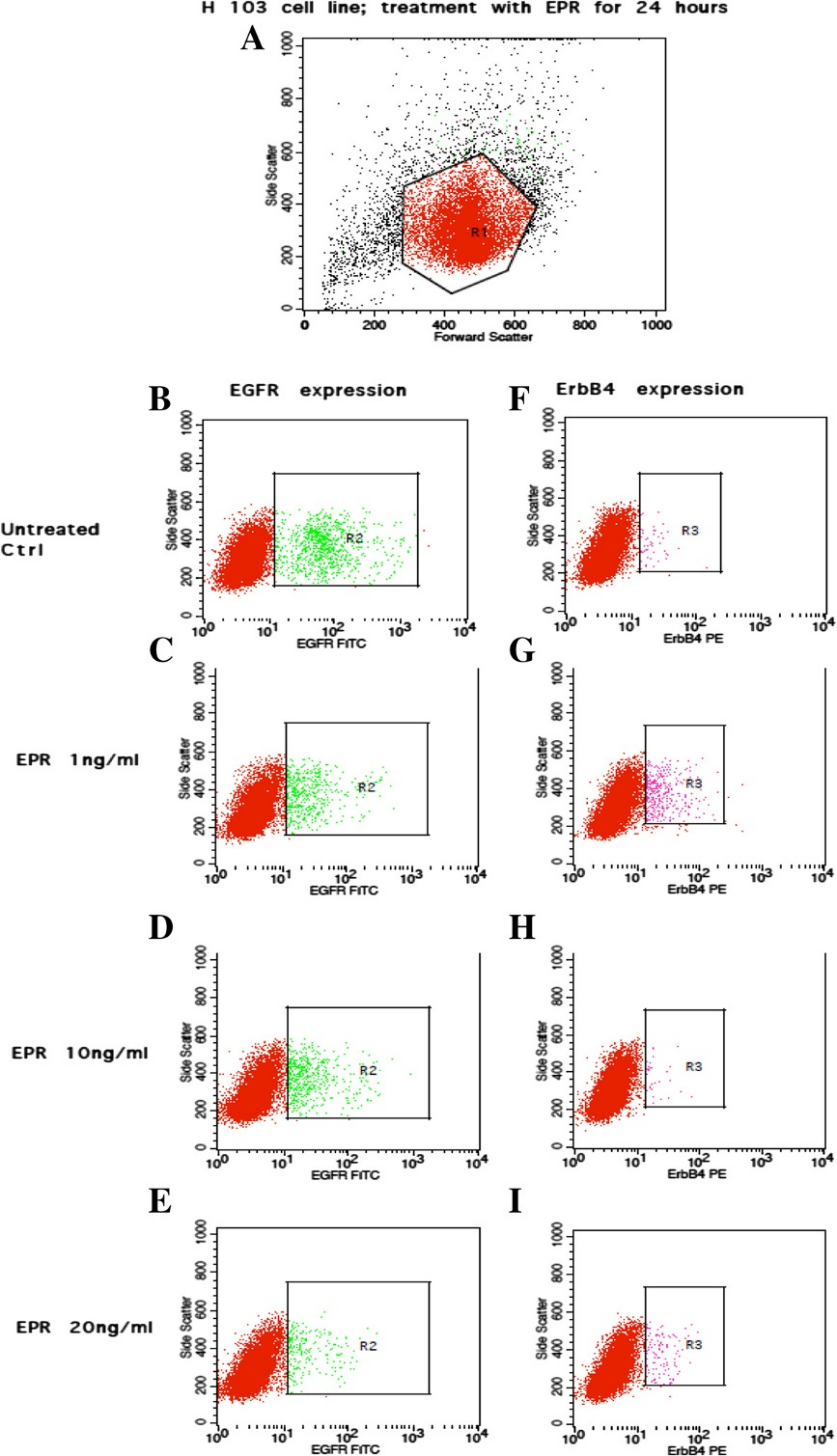
**Flow cytometric output for the EGFR and ErbB4 receptors expression after 24-hour EPR treatment in the H103 cell line.** The outputs for the untreated control are represented by **B** and **F**. **C** and **G**, **D** and **H**, and **E** and **I** represent outputs for cells treated with 1 ηg/ml, 10 ηg/ml, and 20 ηg/ml of EPR respectively. The true percentage of cells and the density of expression of the EGFR or ErbB4 receptors were calculated by subtracting the autofluorescence of the unstained cells (not shown) from the fluorescence of the cells stained with the anti-EGFR-FITC or anti-ErbB4-PE antibodies (B–I) respectively. **A** The R_1_ polygonal box represents the gating of the cells based on the forward and side scatter profiles to exclude cellular debris and doublet cells. A total of 10,000 gated events were acquired for analysis. **B–E** The green dots within the R_2_ boxes represent the fluorescence of cells after staining with anti-EGFR-FITC antibodies. **F–I** The pink dots within the R_3_ boxes represent the fluorescence of cells after staining with anti-ErbB4-PE antibodies.

**Figure 8:**
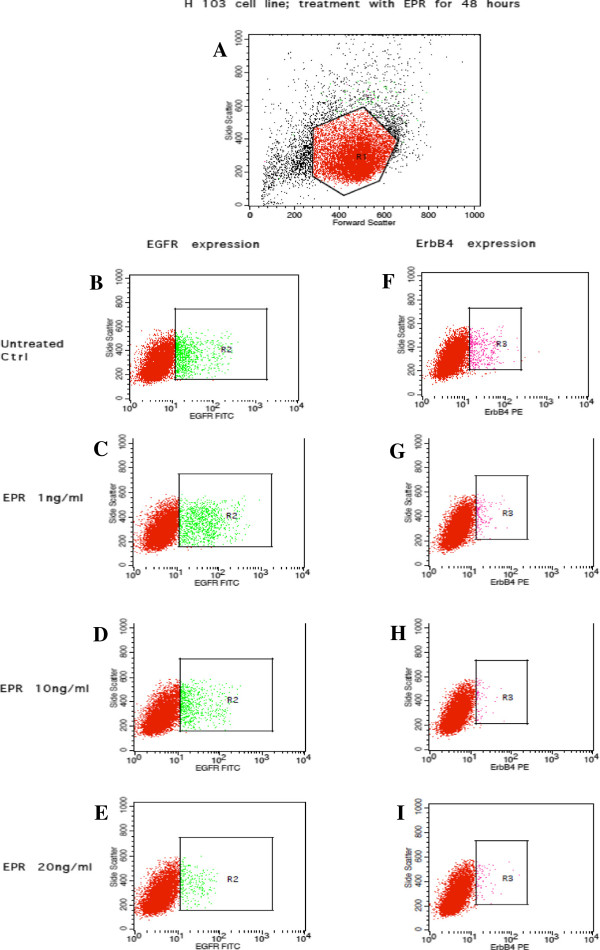
**Flow cytometric output for the EGFR and ErbB4 receptors expression after 48-hour EPR treatment in the H103 cell line.** The outputs for the untreated control are represented by **B** and **F**. **C** and **G**, **D** and **H**, and **E** and **I** represent outputs for cells treated with 1 ηg/ml, 10 ηg/ml, and 20 ηg/ml of EPR respectively. The true percentage of cells and the density of expression of the EGFR or ErbB4 receptors were calculated by subtracting the autofluorescence of the unstained cells (not shown) from the fluorescence of the cells stained with the anti-EGFR-FITC or anti-ErbB4-PE antibodies (B–I) respectively. **A** The R_1_ polygonal box represents the gating of the cells based on the forward and side scatter profiles to exclude cellular debris and doublet cells. A total of 10,000 gated events were acquired for analysis. **B–E** The green dots within the R_2_ boxes represent the fluorescence of cells after staining with anti-EGFR-FITC antibodies. **F–I** The pink dots within the R_3_ boxes represent the fluorescence of cells after staining with anti-ErbB4-PE antibodies.

**Figure 9:**
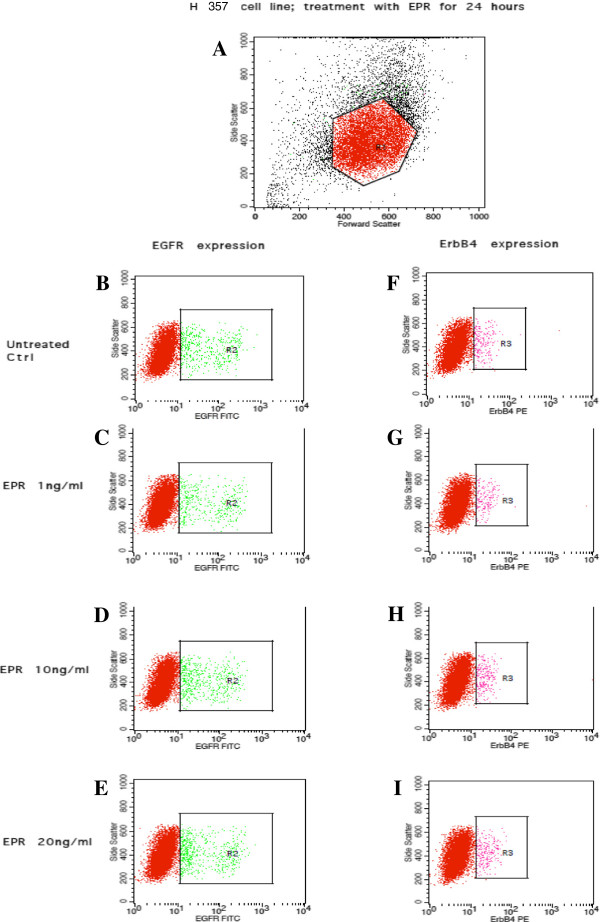
**Flow cytometric output for the EGFR and ErbB4 receptors expression after 24-hour EPR treatment in the H357 cell line.** The outputs for the untreated control are represented by **B** and **F**. **C** and **G**, **D** and **H**, and **E** and **I** represent outputs for cells treated with 1 ηg/ml, 10 ηg/ml, and 20 ηg/ml of EPR respectively. The true percentage of cells and the density of expression of the EGFR or ErbB4 receptors were calculated by subtracting the autofluorescence of the unstained cells (not shown) from the fluorescence of the cells stained with the anti-EGFR-FITC or anti-ErbB4-PE antibodies (B–I) respectively. **A** The R_1_ polygonal box represents the gating of the cells based on the forward and side scatter profiles to exclude cellular debris and doublet cells. A total of 10, 000 gated events were acquired for analysis. **B–E** The green dots within the R_2_ boxes represent the fluorescence of cells after staining with anti-EGFR-FITC antibodies. **F–I** The pink dots within the R_3_ boxes represent the fluorescence of cells after staining with anti-ErbB4-PE antibodies.

**Figure 10:**
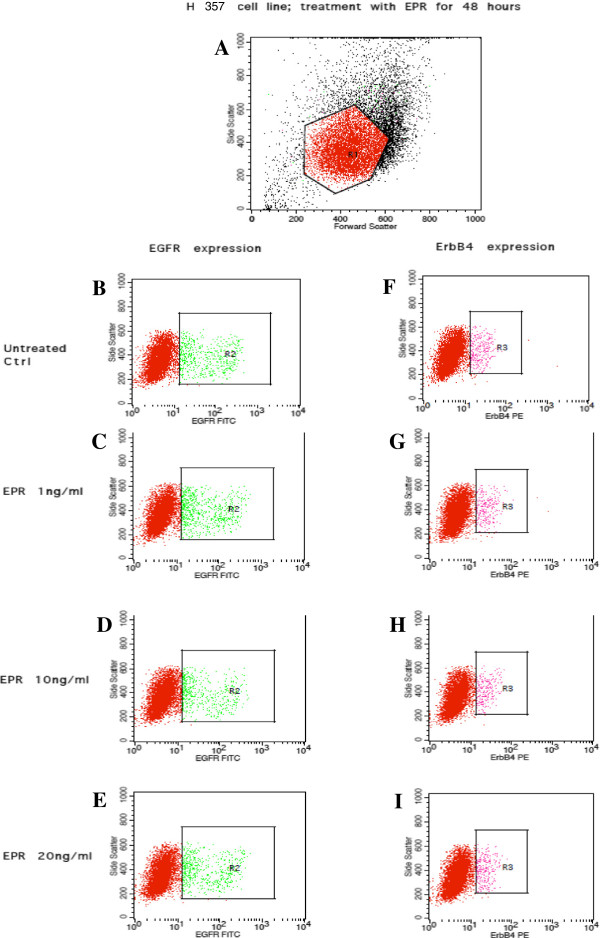
**Flow cytometric output for the EGFR and ErbB4 receptors expression after 48-hour EPR treatment in the H357 cell line.** The outputs for the untreated control are represented by **B** and **F**. **C** and **G**, **D** and **H**, and **E** and **I** represent outputs for cells treated with 1 ηg/ml, 10 ηg/ml, and 20 ηg/ml of EPR respectively. The true percentage of cells and the density of expression of the EGFR or ErbB4 receptors were calculated by subtracting the autofluorescence of the unstained cells (not shown) from the fluorescence of the cells stained with the anti-EGFR-FITC or anti-ErbB4-PE antibodies (B–I) respectively. **A** The R_1_ polygonal box represents the gating of the cells based on the forward and side scatter profiles to exclude cellular debris and doublet cells. A total of 10,000 gated events were acquired for analysis. **B–E** The green dots within the R_2_ boxes represent the fluorescence of cells after staining with anti-EGFR-FITC antibodies. **F–I** The pink dots within the R_3_ boxes represent the fluorescence of cells after staining with anti-ErbB4-PE antibodies.

Figures 7, 8, 9 and 10 show the effects of EPR on the EGFR and ErbB4 receptors expression based on flow cytometric outputs for the H103 cell line and for H357 cell line respectively after 24- and 48-hour treatment respectively. From these outputs, the (i) percentage of cells and (ii) density of expression of EGFR and ErbB4 receptors were determined. In order to assess any changes in receptor expression after EPR treatment, we compared the percentage of expression/mean fluorescence intensity (MFI) of EGFR and ErbB4 between each EPR-treated group, namely 1 ng/ml, 10 ng/ml and 20 ng/ml, with the control.

### (i)Percentage of cells expressing EGFR and ErbB4 receptor

#### H103 cell line

**Figure 11:**
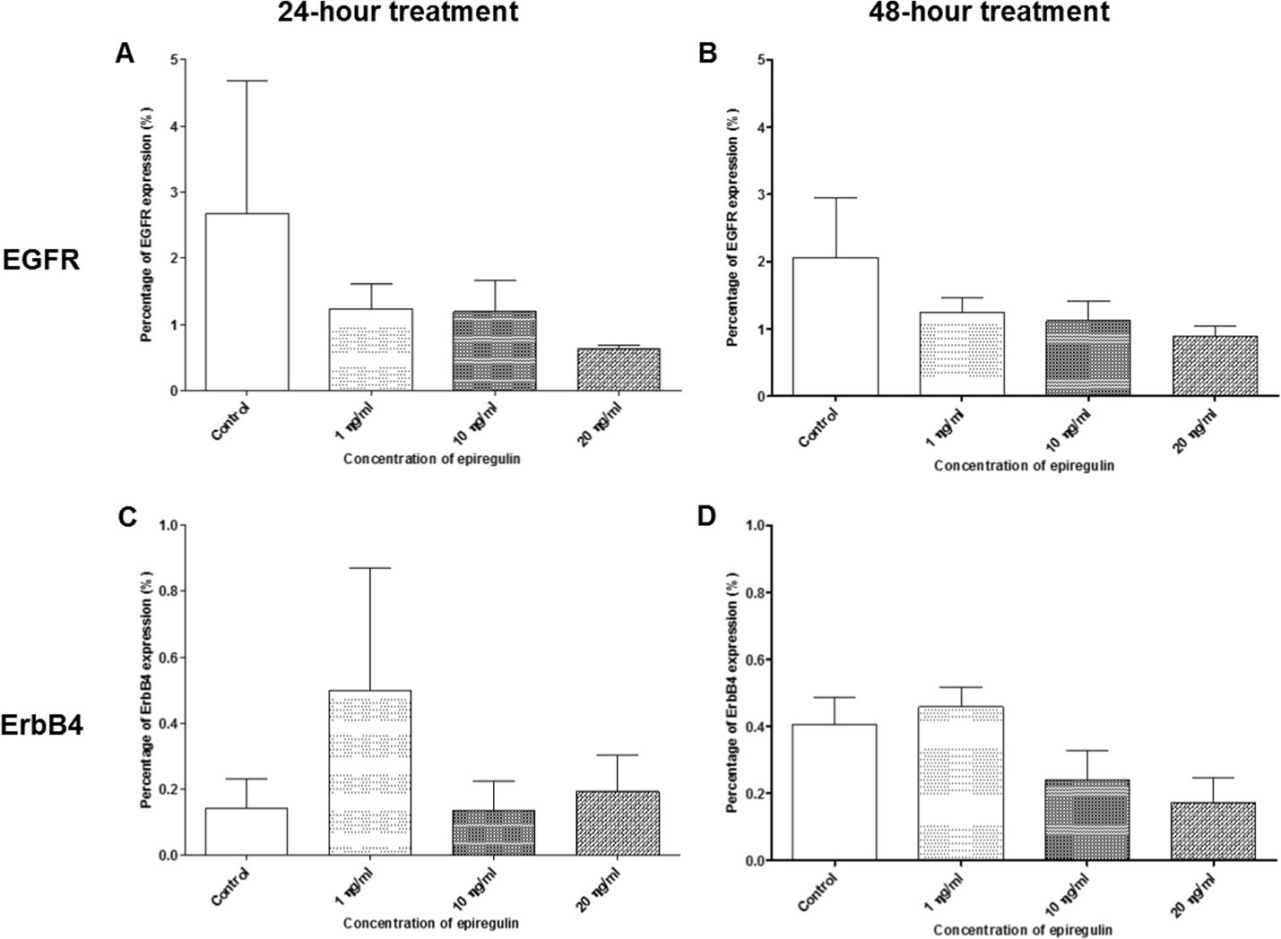
**Percentage of cells expressing EGFR and ErbB4 receptors in the H103 cell line.** The percentage of cells expressing EGFR treated for **A** 24 hours and **B** 48 hours, and the percentage of cells expressing ErbB4 treated for **C** 24 hours and **D** 48 hours.

Figure 11A–B show decreases in the percentage of cells expressing EGFR compared to the control in all treated groups (regardless of the dose of EPR), when EPR was added to the cell line and left for 24 and 48 hours. On the other hand, Figure 11C shows increases in the percentage of cells expressing ErbB4, when 1 ng/ml and 20 ng/ml of EPR (but not 10 ng/ml which showed a decrease) were added to the cell line and left for 24 hours. Similarly, Figure 11D shows an increase in the percentage of cells expressing ErbB4 compared to the control, when 1 ng/ml of EPR (but not 10 ng/ml and 20 ng/ml which showed decreases) was added to the cell line and left for 48 hours. These changes however, were not statistically significant.

#### H357 cell line

**Figure 12:**
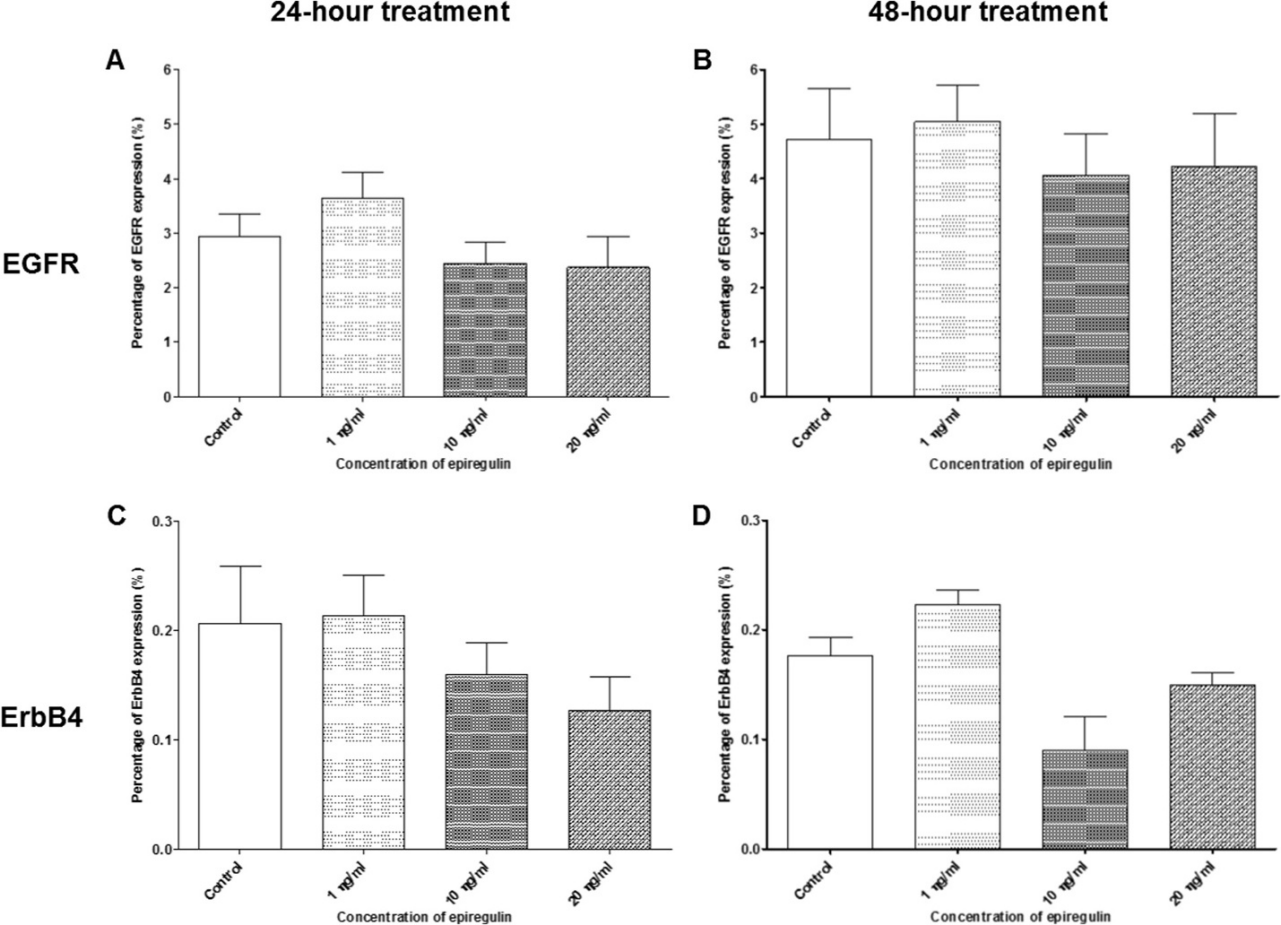
**Percentage of cells expressing EGFR and ErbB4 receptors in the H357 cell line.** The percentage of cells expressing EGFR treated for **A** 24 hours and **B** 48 hours, and the percentage of cells expressing ErbB4 treated for **C** 24 hours and **D** 48 hours.

Figure 12A–B show increases in the percentage of cells expressing EGFR compared to the control, when 1 ng/ml of EPR (but not 10 ng/ml and 20 ng/ml which showed decreases) was added to the cell line and left for 24 and 48 hours. On the other hand, Figure 12C–D show increases in the percentage of cells expressing ErbB4 compared to the control, when 1 ng/ml of EPR (but not 10 ng/ml and 20 ng/ml which showed decreases) was added to the cell line and left for 24 and 48 hours. These changes however, were not statistically significant.

### (ii)Density of receptors: EGFR and ErbB4

#### H103 cell line

**Figure 13:**
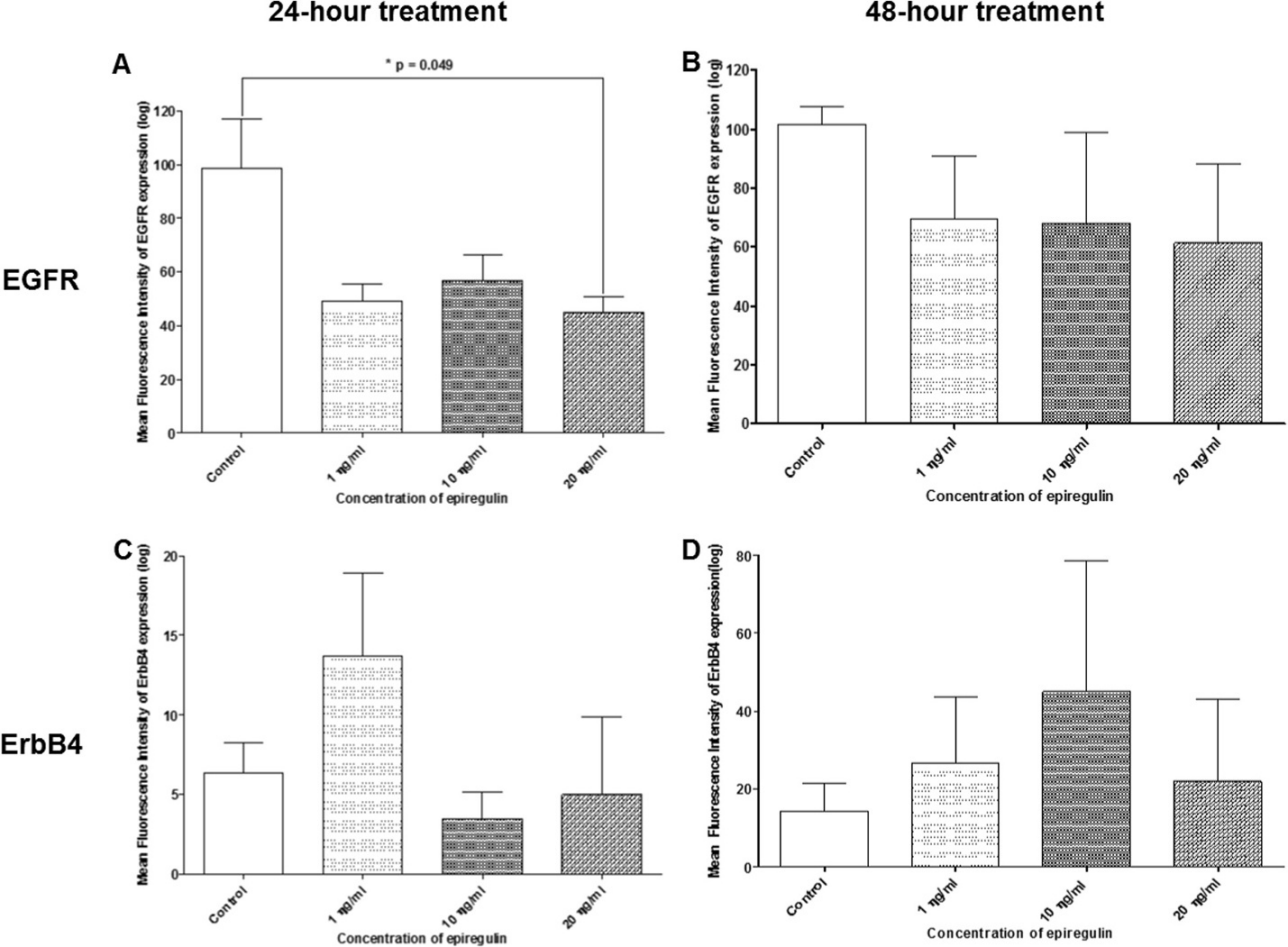
**Density of EGFR and ErbB4 receptors in the H103 cell line.** The density of EGFR receptors treated for **A** 24 hours and **B** 48 hours, and the density of ErbB4 receptors treated for **C** 24 hours and **D** 48 hours. *Represents significant reduction in density of EGFR receptors (student t-test, p-value = 0.049).

Figure 13A–B show decreases in the density of EGFR expression in all treated groups (regardless of the dose of EPR) compared to the control, when EPR was added to the cell line and left for 24 and 48 hours. A significant decrease in the density of EGFR expression was detected (p-value = 0.049) when 20 ng/ml of EPR was added to the cell line and left for 24 hours as shown in Figure 13A. Figure 13C shows an increase in the density of ErbB4 expression compared to the control, when 1 ng/ml of EPR (but not 10 ng/ml and 20 ng/ml which showed decreases) was added to the cell line and left for 24 hours. On the other hand, Figure 13D shows increases in the density of ErbB4 expression in all treated groups (regardless of the dose of EPR) compared to the control, when EPR was added to the cell line and left for 48 hours. These changes other than the one in Figure 13A however, were not statistically significant.

#### H357 cell line

**Figure 14:**
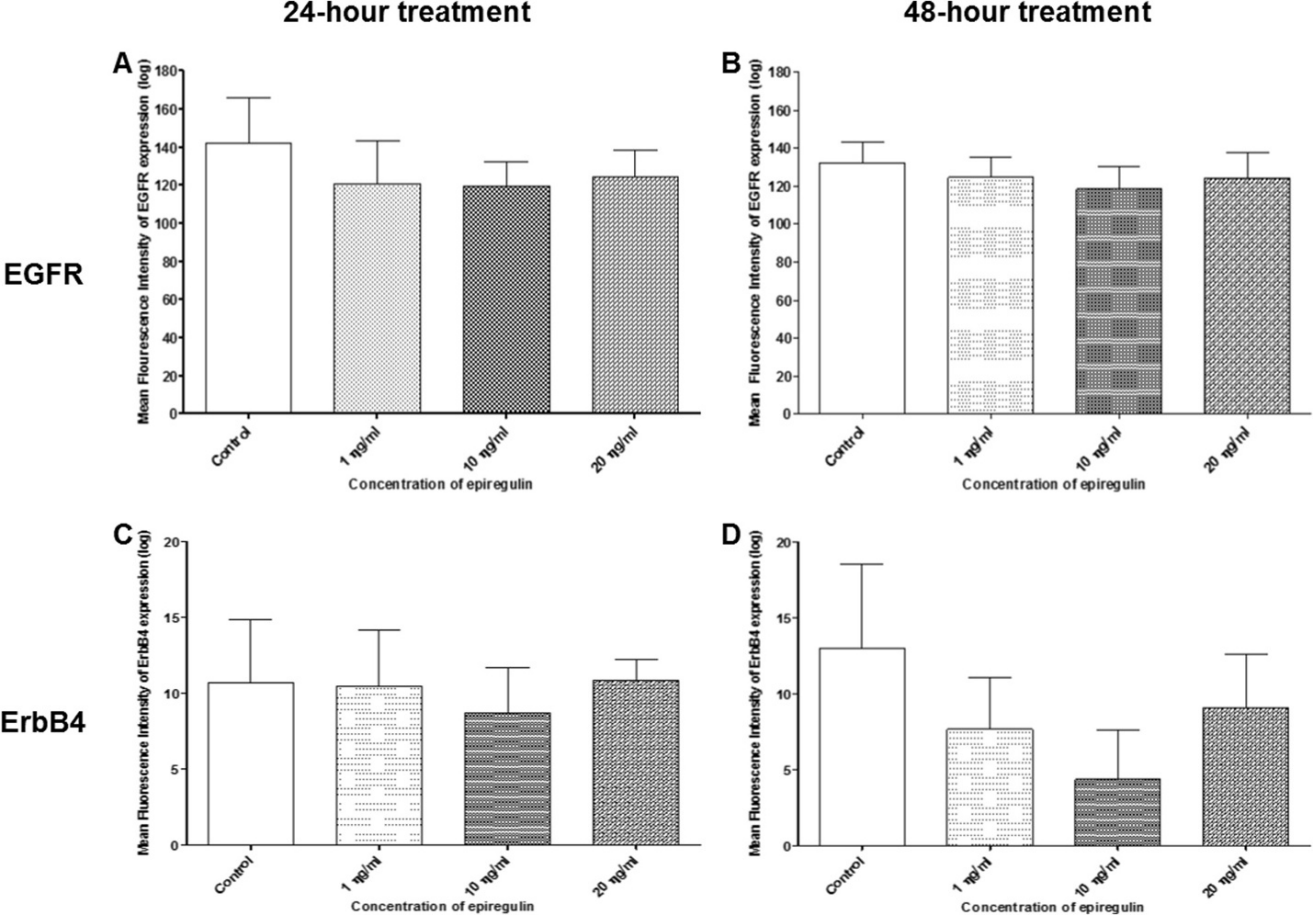
**Density of EGFR and ErbB4 receptors in the H357 cell line.** The density of EGFR receptors treated for **A** 24 hours and **B** 48 hours, and the density of ErbB4 receptors treated for **C** 24 hours and **D** 48 hours.

Figure 14A–B show decreases in the density of EGFR expression in all treated groups (regardless of the dose of EPR) compared to the control, when EPR was added to the cell line and left for 24 and 48 hours. On the other hand, Figure 14C shows an increase in the density of ErbB4 expression compared to the control, when 20 ng/ml of EPR (but not 1 ng/ml and 10 ng/ml which showed decreases) were added to the cell line and left for 24 hours. However, Figure 14D shows decreases in the density of ErbB4 expression in all treated groups (regardless of the dose of EPR) compared to the control, when EPR was added to the cell line and left for 48 hours. These changes however, were not statistically significant.

## Discussion

Up to 90% of oral cancers are OSCCs. Oral cancer is mostly diagnosed by the conventional method of oral inspection and palpation [67–69] but most oral cancers are already in their late stages at the time of detection, resulting in higher patient morbidity and mortality. A great amount of research is being put into identifying suitable biomarkers for earlier detection of oral cancers but existing or newly identified biomarkers are yet to be validated clinically. In this aspect, EPR cannot be ignored as a potential candidate for use as a diagnostic marker and prognosticator although this requires further studies into its role in OSCC and its clinical implications.

Epiregulin was particularly selected for investigation following the recent report by Shigeishi *et al.*[34] who studied EPR mRNA expression in archival material and showed a significant correlation between higher EPR mRNA and poorer survival in OSCC patients. Their study indicated that EPR could play a role in OSCC progression. No other studies have investigated the role of EPR in OSCC cell lines. Thus, the present study has provided an insight into the possible role of EPR in the growth and characteristics of OSCC cells in cell lines, by observing and assessing the changes in cell morphology and proliferation. We investigated whether EPR exerted its effect via the regulation of two types of EGF receptors i.e. EGFR or ErbB4, since EPR has been known to bind directly to these receptors [54]. In order to achieve that, it was therefore important to establish whether the H-series cell lines express these receptors through screening.

Prime *et al.*[66] showed through immuno-staining and radio-ligand binding assays that all the H-series cell lines expressed EGFR. The results of the present study confirm the positivity of EGFR expression in all the H-series cell lines. In addition to EGFR, the present study also demonstrated that almost all the H-series cell lines expressed ErbB4. The H357 ErbB4 expression was negative due to very minimal detection of its expression (percentage of ErbB4 expression < 0.01%). The present study is the first to demonstrate the expression of ErbB4 in the H-series OSCC cell lines and would be useful for future studies using this cell line.

The ability of EPR to cause morphological changes in OSCC (especially in dedifferentiation) is clinically important. Oral squamous cell carcinomas are mostly well-differentiated and moderately- to well-differentiated [2, 70] with lower differentiation associated with increased tumour aggressiveness. Poor patient outcome in terms of tumour recurrence, metastasis, and worst disease-free survival is associated with OSCC tumours that are poorly-differentiated [71–73]. In the present study, treatment with EPR produced no changes in the morphology of the H103 and H357 cell lines. This may be due to the tissue expression of EPR predominating in the macrophages and placenta but not particularly so in other tissues [65], thus suggesting that differentiating functions of EPR lies mainly in reproductive and immune physiology. Although other studies [57, 60, 74] have shown that EPR induced morphological changes in ovarian granulosa cells, epidermal keratinocytes and vascular smooth muscle cells, it is possible that EPR does not have differentiating functions in oral cells. The lack of change in the morphology of the H103 and H357 cell lines may be due to the short treatment periods of these cell lines with EPR over 24 and 48 hours. Previous studies have used cells from primary cultures and therefore the findings in the present study are not comparable. Once successfully cultivated *in vitro*, cells from primary cultures become cell strains which possess characteristics not seen in cell lines such as maintenance of the diploid karyotype, retention of histotypical differentiation, and having similar cell morphology to primary tissue [75, 76]. If this fact holds true in the H103 and H357 cell lines, it could be that they have lost their ability to differentiate. Last but not least, the absence of interaction with other cell types such as neighbouring fibroblasts as per *in vivo* could directly or indirectly mediate the effects on EPR on OSCC cell differentiation as demonstrated in studies of other tissues [77, 78].

The proliferativity of OSCCs has been linked to higher tumour-node-metastasis (TNM) grading, poorer prognosis, and tumour differentiation with poorer differentiation associated with higher proliferativity as shown in a cytokinetic study in OSCCs [79]. An immunohistochemical study on archival OSCC specimens established an association between higher OSCC proliferative index with older patients, late clinical staging, larger tumour size, nodal metastasis, and distant metastasis [80]. Shirakata *et al.*[60] and Morita *et al.*[62] demonstrated that EPR cause a logarithmic increase in the number of cells in human epidermal keratinocytes and human corneal epithelial cells and these increases were dose-dependent. Zhuang *et al.*[55] reported that EPR enhanced proliferation of rabbit RPTCs. These studies demonstrated that an optimal EPR dose of 10 ng/ml with an effective dose up to 20 ng/ml was essential for enhanced proliferation. Bringing together the results of the cell counts and BrdU proliferation assays, the present study demonstrated that EPR did stimulate marginal increases in cell proliferation although these findings were not statistically significant. This phenomenon could be due to several reasons, the first being that the concentrations of EPR of ≤ 20 ng/ml used may be too low to elicit a significant cellular response in OSCC cell lines. Sasaki *et al.*[81] and Zhu *et al.*[82] showed that EPR was able to significantly promote proliferation of rabbit gastric cancer cells and pancreatic cancer cell lines respectively at concentrations up to 100 ng/ml. The marginal increases may also be attributed to the different cell types i.e. epidermal keratinocytes or RPTCs which respond differently to EPR compared to OSCC cells.

Other than differential responses, the short treatment periods of 24 and 48 hours could be the other contributing factors for the marginal increases in cell proliferation. Similar studies by Morita *et al.*[62], Zhang *et al.*[83], and Lindvall *et al.*[84] employed longer treatment periods of between six to twelve days. Previous studies have also used different techniques to measure cell proliferation such as protein and dye reduction assays which have different sensitivities and specificities. This study has demonstrated that EPR may have the potential for promoting greater OSCC proliferation if EPR concentrations or treatment periods were increased.

Binding of EGF family ligand(s) and activation of their respective receptor(s) have been reported to lead to the internalisation of the ligand-receptor complex prior to lysosomal targeting and degradation (reviewed in reference 25). This process will subsequently reduce the cell surface expression of the affected receptor(s). With this, it is plausible that EPR could also down-regulate the expression of EGFR and ErbB4. In the present study, the only significant reduction detected was the density of EGFR expression in the H103 cell line which occurred at the EPR concentration of 20 ng/ml after 24 hours of treatment. This finding concurred with Citri and Yarden’s model of receptor regulation [25]. This significant reduction could also be explained by EGFR homo-dimer formation which occurs preferentially at high ligand concentrations and which are internalised more efficiently than EGFR hetero-dimers [85]. However, it is yet unclear why within the same cell line there was no significant reduction in the density of EGFR expression after the 48-hour treatment. It could be that EPR is rapidly and extensively depleted by EGFR uptake with subsequent internalisation and degradation, resulting in reduced concentration of EPR after 48 hours [14, 64]. This lowered concentration of EPR would then be unable to induce any significant down-regulation of EGFR.

The minimal changes in EGFR expression may be due to the interference of EGFR internalisation [86] and the associated limitation in the cellular components mediating receptor down-regulation, thus making it a saturable process at the level of internalisation [87] as well as at the level of targeting of ligand-receptor complexes to lysosomes for degradation [88]. In other words, the capacity of the intracellular molecular components mediating receptor down-regulation defines the maximum amount of active receptors that can be down-regulated and degraded at any one time, regardless of the number of ligand-activated EGFR. In addition, there may also be a significant extent of receptor hetero-dimerisation which slowed post-endocytic trafficking of internalised receptors. Lenferink *et al.*[89] and Worthylake *et al.*[90] demonstrated that EGFR-ErbB2 hetero-dimers slowed internalisation and targeting of ligand-receptor complexes to lysosomes. It must be noted that EPR possess the capacity to activate almost all possible hetero-dimeric EGF receptor complexes [56]. In the present study, the ErbB2 receptor was not investigated and the possibility of its influence on regulation of EGFR expression after EPR stimulation thus, cannot be excluded. Lenferink *et al.*[89] demonstrated that EGF stimulation rapidly diminishes EGFR expression in contrast to TGF-α which caused reappearance of EGFR presumably due to recycling of endocytosed receptors. These studies indicate that hetero-dimerisation with another EGF family receptor slows the down-regulation of EGFR expression and the type of EGF family ligand that binds to EGFR also dictates the regulation of EGFR expression.

There were no significant changes in the ErbB4 expression after EPR treatment in both the H103 and H357 cell lines in the present study. This confirms that the H357 cell line is truly negative for ErbB4, verifying the results of the initial screening for ErbB4 expression. Evidence has shown that ErbB4 receptor is deficient in several components of the down-regulatory system and that it behaves rather differently in comparison to EGFR. Citri and Yarden [25] reviewed that unlike EGFR, ErbB4 does not possess the capacity to directly recruit certain intracellular elements involved with receptor internalisation and degradation. Several studies postulated that regulation of ErbB4 expression is ligand-independent [91, 92] and may explain why EPR did not induce any significant changes in ErbB4 expression in the present study. The non-significant changes in EGFR and ErbB4 expression after EPR stimulation in both H103 and H357 cell lines may be due to the rate of receptor recycling exceeding that of receptor internalisation. In addition, intracellular EGFR and ErbB4 receptors stored within vesicles in the cellular cytoplasm might have been directed to the cell surface during this recycling process resulting in a surplus expression of receptors in cells treated with EPR compared to the untreated control [25].

Based on previous studies, there is cumulative evidence that the aggressiveness of OSCC is related to the patients’ age i.e. the younger the patient, the worse the prognosis [93–96]. Interestingly, in the present study, the 5-year survival of the patients as dictated by the STNMP grade is the same in both the H103 and H357 cell lines despite the age differences (Table 3).

**Table 3:** **Summary of the clinicopathological characteristics and receptor expression of the H103 and H357 cell lines**

Cell line	Age	STNMP grade ^a^	EGFR expression	ErbB4 expression
H103	32	I	Significantly reduced in density after 24 hour treatment.	No significant changes.
H357	74	I	No significant changes.	No significant changes.
				Confirmed to be negative for ErbB4.

^a^STNMP grade: prognostic indicator for OSCC with 51.5%, 40.7%, 21.6% and 8.3% 5-year survival for patients with a stage I, II, III or IV tumours, respectively.

In terms of clinical implications of EGFR expression on patient survival, a higher EGFR expression was associated with increasing age and poorer survival rates. This association was frequently found in breast [97, 98] and colorectal cancers [99, 100]. A study by Thomas *et al.*[101] on archival OSCC specimens of young adults aged between 18 to 40 years showed that low EGFR expression conferred a 100% five-year survival rate while high EGFR expression reduced the survival rate to 81.1%. Upon this basis, it can be inferred that the good survival rate in the younger patient (from which the H103 cell line originated) (Table 3) could possibly be attributed to the ability of the EGFR receptor to be significantly down-regulated upon ligand binding. There was however, no correlation between ErbB4 expression and OSCC prognosis. This concurred with findings from several other similar studies looking at other epithelial cancers [102–104]. This shows that ErbB4 might not be an independent predictor for survival of OSCC patients. Despite all these studies, it must be noted that there have been conflicting reports on the correlation between the age of the patient with survival, and the association between survival and EGFR and ErbB4 expression [99, 100, 105–112]. In one study, higher expression of ErbB4 has been linked to better prognosis [113].

## Conclusions

Epiregulin could significantly decrease (down-regulate) EGFR expression but not the ErbB4 receptor in OSCC cell lines. However, it does not significantly affect the cell morphology in OSCC cell lines nor does it significantly enhance OSCC cell proliferation.

Whilst it is possible that when EPR binds to EGFR, it induces down-regulation of this receptor which leads to signal attenuation for differentiation and proliferation of OSCC cells as seen in the H103 cell line. This attenuation in differentiation and proliferation could explain why there were no significant changes in cellular morphology and proliferation in the present study. With reference to the clinicopathological origins of the H103 and H357 cell lines, it is also possible that the good survival rate for the younger patient may be attributed to this phenomenon. The ability of EPR to significantly down-regulate EGFR in the H103 cell line after 24 hours of treatment but not after 48 hours suggests that signal attenuation may be achieved by other means. As for the H357 cell line, no significant changes in morphology and proliferation were seen, suggesting that there is an inherent heterogeneity in biological responses even between cells (OSCC) of the same type despite no significant changes in EGFR expression. On the other hand, the ErbB4 receptor most likely did not play a role in OSCC cellular differentiation and proliferation. The results of the expression of ErbB4 after EPR treatment in the present study substantiate the fact that the regulation of ErbB4 expression may be ligand-independent.

Despite the previous and present investigations, it must be noted that the level of EGFR and ErbB4 receptors expression might not imply a cause-and-effect relationship with OSCC progression.

## Materials and methods

### Cell culture and material

Seven OSCC cells lines, namely H103, H157, H314, H357, H376, H400, and H413 were obtained for the screening of suitable cell lines to be incorporated in the studies. All cell lines were cultured in Dulbecco’s modified Eagle’s medium (DMEM) nutrient mixture F-12 Ham supplemented with 10% foetal bovine serum (FBS), 0.5 μg/ml hydrocortisone, and 1% of penicillin-streptomycin at 37°C in 5% carbon dioxide (CO^2^) tissue culture incubator. The cells were split at a 1:3 ratio and passaged every three days upon confluence. The cell lines were treated for one cycle with 2 μg/ml of gentamicin replacing penicillin-streptomycin, added directly into complete medium. Thereafter, the cells were maintained in antibiotics-free medium for all downstream experiments.

### Selection of cell lines for downstream experiments

All the H-series cell lines were characterised based on the clinicopathological characteristics of the patients where the cell lines were derived from, and also on their receptors, particularly EGFR and ErbB4. Both characterisations were done simultaneously for the selection of suitable cell lines for downstream experiments in the present study.

### Clinicopathological considerations

The clinicopathological characteristics of the patients from which the H-series cell lines were derived are shown in Table 1. [66]. The criteria for selecting the cell lines were age, sex, and STNMP grade of the patients.

### EGFR and ErbB4 receptors screening

For surface receptor analysis of all cell lines, cells were trypsinised using 0.25% of trypsin-ethylenediaminetetraacetic acid (EDTA) for 5 minutes and subsequently inhibited with complete culture medium. The cells (1 × 10^6^) were washed with phosphate-buffered saline (PBS) and blocked with 10% of FBS in PBS for 10 minutes prior to incubation with antibodies. The incubation of the cells with 10% of FBS in PBS functioned as a blocker to inhibit the unspecific Fc receptors of the cells. Subsequently, the cells were incubated with anti-EGFR-fluorescein (FITC) and anti-ErbB4-phycoerythrin (PE) antibodies respectively in staining buffer containing 1% of bovine serum albumin (BSA) in PBS for 30 minutes at 4°C. The cells were washed twice with PBS to remove excess antibodies and suspended in 500 μl of PBS prior to analysis. Flow cytometry was performed using a FACSCalibur flow cytometer and data obtained was analysed using the CellQuest Pro software. Cells were gated on the basis of forward and side scatter profile to exclude cellular debris and doublet cells. A total of 10, 000 events were taken for analysis. A positive (+) EGFR or ErbB4 expression is defined as a percentage expression of ≥ 0.01% and a negative (-) expression is defined as a percentage expression of < 0.01%.

### Epiregulin treatment

Two cells lines, H103 and H357, were cultured overnight in complete culture medium before EPR treatment. The test group of cells were treated with 1 ng/ml, 10 ng/ml or 20 ng/ml of EPR each time, added into the complete culture medium for 24 and 48 hours respectively. The control group contained complete culture medium without addition of EPR.

### Morphology observation

Control and treated cells were observed under low magnification (20×) phase contrast microscopy for the identification of the cells’ morphology and morphology changes (if any) upon EPR treatment. The morphological characteristics assessed include size, shape, nuclear and cytoplasmic changes.

### Cell count for viability

Cell counts for control and treated cells were performed using standard trypan blue exclusion method using a haemocytometer. The experiments were conducted in triplicates and performed thrice to confirm the reproducibility.

### BrdU proliferation assay

Cells were plated into 96-well plates in 200 μl of complete culture medium. The plates were then incubated overnight at 37°C in 5% CO^2^. The medium was then removed and exchanged with culture medium containing the various concentrations of EPR. Control cells were maintained in complete culture medium.

Cell proliferation was monitored at 24 and 48 hours after commencement of EPR treatment by adding 20 μl of BrdU labelling solution into each well of the 96-well plate and incubated for another 4 hours at 37°C in 5% CO^2^. Following incubation, the cells were washed twice with complete culture medium. The cells were then fixed with 70% ethanol in 0.5 M hydrochloric acid (HCl) for 30 minutes at -20°C. The cells were then washed thrice with complete medium before incubation with 100 μl of nucleases for 30 minutes at 37°C in a water bath. The cells were subsequently washed thrice with complete medium and incubated with 100 μl of anti-BrdU-peroxidase (POD), Fab fragments for 30 minutes at 37°C. The cells were finally washed thrice with PBS and incubated with 100 μl of peroxidase substrate at room temperature for 30 minutes prior to analysis. The plate was read on a microplate reader at a wavelength of 405 nm. The experiments were conducted in triplicates and performed thrice to confirm the reproducibility.

### Surface receptor analysis for EGFR and ErbB4

The surface receptor analysis was performed using the methods as described under ‘EGFR and ErbB4 receptors screening’. The MFI of EGFR and ErbB4 was used to determine the expression density of these receptors (the amount of receptors over the number of gated cells) on the treated cells when compared to the control cells. For the percentage of expression and MFI changes after treatment with EPR, 1 × 10^6^ of EPR-treated and control cells were obtained using the methods as described earlier. The quantitative flow cytometric analysis for the percentage of expression of anti-EGFR- and anti-ErbB4-labelled cells was calculated by counting labelled cells which exceeded the upper limit of the auto-fluorescence of unlabelled cells. The presented MFI of labelled cells was calculated by subtracting the auto-fluorescence intensity of the unlabelled cells within the same sample. The experiments were conducted in triplicates and performed thrice to confirm the reproducibility.

### Statistical analysis

All data were represented as mean values ± standard error of mean. Results were analysed using SPSS version 18. Because the number of samples is only three in the control group as well as each of the EPR-treated group, namely 1 ng/ml, 10 ng/ml and 20 ng/ml, we believe that the sample is normally distributed and hence the student t-test was used to analyse any two independent groups within the study. The level of significance for all comparisons was set at 0.05 and statistically significant results (p < 0.05) were highlighted.

## Authors’ information

Darren Chyi-Hsiang Kong

BMedSc (IMU)

Kenneth Yee Choy Chew

BSc Biomed (UPM)

Assistant Lecturer of School of Science, Monash University Malaysia (MUM)

Associate Professor Dr. Eng Lai Tan

BSc (Murd), M Biotech (UM), PhD (UM)

Associate Professor of School of Pharmacy, International Medical University (IMU)

Professor Suan Phaik Khoo

BDS (UM), MSc (Lond), FFDRCSI (Oral Med), Cert. Immunol (Lond), PhD (National University of Singapore)

Professor of Oral Pathology & Associate Dean of Oral Sciences, School of Dentistry, International Medical University (IMU)

## Abbreviations

OSCC: Oral squamous cell carcinoma
EGF: Epidermal growth factor
TGF-α: Transforming growth factor-alpha
AR: Amphiregulin
HB-EGF: Heparin-binding-epidermal growth factor
BTC: Betacellulin
EPR: Epiregulin
EPG: Epigen
NRG: Neuregulin
EGFR: Epidermal growth factor receptor
MMP: Matrix metalloproteinase
MAPK: Mitogen-activated protein kinase
ERK: Extracellular signal-regulated kinase
PI3K: Phosphatidylinositol-3-kinase
VSMC: Vascular smooth muscle cell
RPTC: Renal proximal tubule cell
MFH: Malignant fibrous histiocytoma
mRNA: Messenger RNA
STNMP: Site-size-nodal metastasis-distant metastasis-pathology
BrdU: 5-bromo-2’-deoxy-uridine
MFI: Mean fluorescence intensity
TNM: Tumour-node-metastasis
DMEM: Dulbecco’s modified Eagle’s medium
FBS: Foetal bovine serum
CO^2^: Carbon dioxide
EDTA: Ethylenediaminetetraacetic acid
FITC: Fluorescein
PE: Phycoerythrin
BSA: Bovine serum albumin
HCl: Hydrochloric acid
POD: Peroxidase
PBS: Phosphate-buffered saline.
